# Application of Biosurfactants in Medical Sciences

**DOI:** 10.3390/molecules29112606

**Published:** 2024-06-01

**Authors:** Xiaoyan Wang, Jiachen An, Tianyu Cao, Mingmin Guo, Fu Han

**Affiliations:** School of Light Industry Science and Engineering, Beijing Technology and Business University, Beijing 100048, China; 2130031031@st.btbu.edu.cn (X.W.); 2130032051@st.btbu.edu.cn (J.A.); 2230301018@st.btbu.edu.cn (T.C.); 2330302074@st.btbu.edu.cn (M.G.)

**Keywords:** biosurfactants, antibacterial, antiviral, carrier, gene, immunomodulation

## Abstract

Biosurfactants derived from microorganisms have attracted widespread attention in scientific research due to their unique surface activity, low toxicity, biodegradability, antibacterial properties, and stability under extreme conditions. Biosurfactants are widely used in many fields, such as medicine, agriculture, and environmental protection. Therefore, this review aims to comprehensively review and analyze the various applications of biosurfactants in the medical field. The central roles of biosurfactants in crucial medical areas are explored, like drug delivery, induction of tumor cell differentiation or death, treating bacterial and viral effects, healing wounds, and immune regulation. Moreover, a new outlook is introduced on optimizing the capabilities of biosurfactants through modification and gene recombination for better use in medicine. The current research challenges and future research directions are described, aiming to provide valuable insights for continuous study of biosurfactants in medicine.

## 1. Introduction

Biosurfactants are remarkable substances derived from microorganisms such as bacteria, yeast, or fungi, and are garnering increasing attention due to their notably surface activity and biocompatibility [1]. These unique characteristics enable them to serve significant roles in a multitude of areas, the most promising of which lies in the medical realm. In recent years, the potential of biosurfactants in medicine has unveiled immense opportunities and challenges. In particular, their prospective uses as drug carriers [2], antibacterial agents [3], antiviral agents [4], and therapeutic agents in tumor treatments [5] are opening new avenues for the advancement of healthcare.

A more comprehensive understanding of biochemical properties of biosurfactants has been gained through extensive investigations of microbial strains and gene encoding [6]. Additionally, genetic editing techniques can be employed to modify and reorganize these microorganisms. As a result, the yield of biosurfactants has been substantially increased [7] and the production costs reduced [8]. This approach also enables heterologous gene expression of biosurfactants using low-toxicity strains [9]. This advance is opening new doors in the field of biomedicine. Nonetheless, the research on applications of biosurfactants as drug carriers and their functions in antibacterial, antiviral, and tumor therapies is still in the early stages. This vast field, filled with unknown potential, inspires us to conduct further and more systematic studies. The studies on biosurfactants in immune regulation [10] and wound healing [11] are advancing, with particular interest in disentangling their underlying mechanisms. Notably, owing to their remarkable biochemical properties, biosurfactants have emerged as a popular research topic in the functional particle modification field [12], demonstrating tremendous potential for combining theory with practice that could pave the way for a plethora of innovative breakthroughs.

This review seeks to extensively outline biosurfactants and their emerging role in medicine. Even though Saimmai [13] and Chiara [14] have extensively described biosurfactants in a medical context, there is still a deficiency in the systematic categorization of their applications and effects within the various disciplines of medical science. Addressing this deficiency, this review summarizes the applications of biological surfactants in multiple medical fields in Table 1, and analyzes them in detail in Section 3. The goal is to promote a quick and thorough understanding of the importance of biological surfactants in these areas. Additionally, this review emphasizes the significant progress made by genetic engineering in the production of biological surfactants, and discusses in detail the wide-ranging medical applications of biological surfactants. Finally, it examines the existing challenges in the field of medical biological surfactants and suggests future research directions, aiming to become an indispensable resource for medical professionals and researchers.

## 2. Biosurfactants: Properties, Synthesis and Classification

### 2.1. Properties of Biosurfactants

Biosurfactants are secondary metabolites of microorganisms with unique surface-active properties. Structurally, these molecules strike a balance between hydrophilicity and hydrophobicity, which helps to reduce the surface tension. Biosurfactants differ from chemical surfactants as they exhibit more polar mosaic distributions and often have branched or cyclic structures. As a result, biosurfactants are more inclined to insert into the phospholipid bilayer through membrane interactions [41]. Biosurfactants have the following characteristics:

(I) Self-assembly: After reaching certain conditions, biosurfactants have the ability to self-assemble into structures known as micelles or vesicles [42]. These self-organized formations enhance the stability of biosurfactants in aqueous environments. By encapsulating drugs, compared to those dispersed in the solution, these formations facilitate accurate drug delivery through the bloodstream to the disease sites, thereby enhancing the therapeutic effect [16]. Furthermore, the encapsulated drugs in micelles or vesicles contribute to the stability of drugs within the body, thus improving their bioavailability [43]. Therefore, the self-assembly behavior of biosurfactants can act as components of drug carriers, effectively augmenting the efficacy and safety of drugs. A comprehensive explanation of this is provided in the third section of this article.

(II) Interaction with cell membranes: Surfactants can adsorb on the cell membrane via electrostatic, van der Waals, hydrogen bond, and hydrophobic forces. The interaction between biosurfactants and cell membranes has significant implications across several facets: (i) Alter the physical properties of the cell membrane: Biosurfactants can alter the fluidity and stability of the cell membrane, thereby affecting its elasticity and permeability [44]. (ii) Affect the biological functions of the cell membrane: Biosurfactants can influence the activity of ion channels [45] and transport proteins on the cell membrane [46], thereby altering the exchange of substances inside and outside the cell and signal transduction. (iii) Deposition on the surface of the cell membrane: Biosurfactants can form monolayers or bilayers on the surface of the cell membrane [47], changing the cell surface characteristics, which may affect functions such as cell adhesion, coagulation, and antigen recognition [48]. (iv) Damage the cell membrane: Under certain conditions, biosurfactants may inflict destructive effects on the cell membrane, which is quite essential in certain antimicrobial and anti-inflammatory applications. The insertion of biosurfactants into cell membranes disrupts lipid distribution, leading to curvature changes or even micellization [49]. This could cause partial dehydration of the membrane and, in extreme cases, the formation of membrane defects or pores [50]. The damage inflicted by biosurfactants on cell membranes exhibits a concentration-dependent characteristic. For instance, lipopeptide biosurfactants only induce pore formation on the lipid bilayer at moderate concentrations. At high concentrations, they act as detergents, leading to cell membrane inactivation and subsequent dehydration of phospholipids [26]. Consequently, biosurfactants can serve various medical purposes, leveraging their membrane-damaging propensities to attain antibacterial and antiviral effects, or utilizing their membrane fusion capabilities to enhance drug delivery efficiency. Biosurfactants have key properties affecting their interaction with membranes, such as the existence of sugar moieties [51], the number of unsaturated bonds [52], chain length [53], and acetylation level [54]. These aspects help to explain the complex and dynamic interaction between biosurfactants and membranes.

(III) Promote the production of cytokines: Firstly, they can alter the characteristics and behavior of the cell membrane, such as changing membrane fluidity, tension, and charge, thereby promoting cell attachment, growth, and the production of cytokines. Secondly, the structure of glycolipid biosurfactants is similar to the static components of cell membranes; biosurfactants can regulate the activity of cell signaling pathways by interacting with cell membranes, which further affects the synthesis and release of cytokines. Moreover, some biosurfactants themselves might have direct immunomodulatory activity, stimulating immune cells to produce and release cytokines. MEL-A is one such significant biosurfactant. Studies revealed that MEL-A not only has high affinity to immunoglobulins, but also exhibits high affinity to Concanavalin A (Con A). It is worth noting that although Con A is primarily present in jack-bean, its effect on mammalian cells has attracted widespread attention and it is commonly used in studies related to immunology and virology [55]. Through these actions, biosurfactants like MEL-A profoundly influence cell functions, including immune responses, and play crucial roles in many biomedical fields.

In conclusion, biosurfactants can form micelle or vesicle structures, interact closely with cell membranes, and bind specifically to cells. These characteristics enable biosurfactants to have enormous potential in drug delivery, antibacterial and antiviral applications, immune regulation, and more. Biosurfactants can also affect the production of cytokines, making them highly promising in immune regulation and inducing tumor cell differentiation or death.

### 2.2. Classification, Bacterial Strains, and Responsible Genes of Biosurfactants

**Figure 1:**
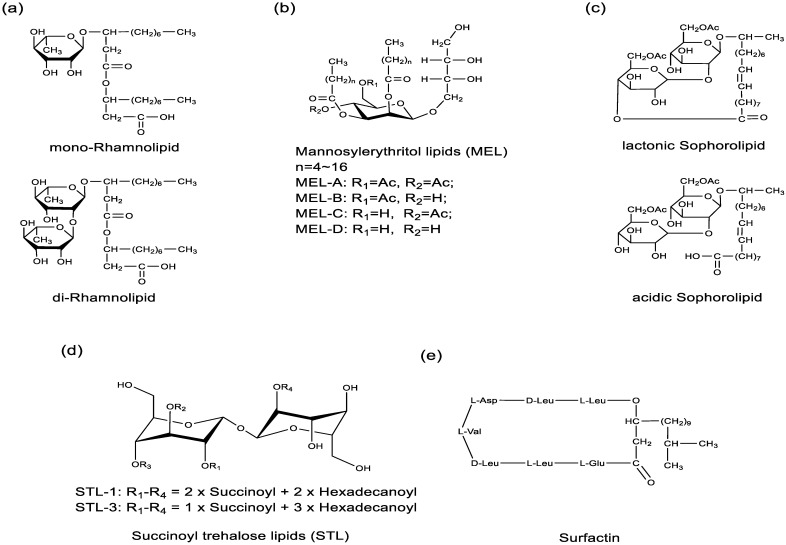
Chemical structure of (**a**) mono- and di-rhamnolipids, (**b**) mannosylerythritol lipids, (**c**) lactonic and acidic form sophorolipids, (**d**) succinoyl trehalose lipids, (**e**) surfactin.

Biosurfactants are metabolic products of microorganisms. Biosurfactants can be classified as glycolipids, lipopeptides, neutral lipids, phospholipids, or fatty acids, based on their chemical structures and microbial origin. The types and quantities of biosurfactants produced by microbes are closely related to their growth environment [56] and nutritional conditions [57]. Therefore, exploring the growth environment and nutritional conditions of microorganisms is also a popular research topic for expanding the yield of target biological surfactants [58]. Currently, the majority of biosurfactant studies concentrate on complex bioactive agents like glycolipids and lipopeptides, whereas there are fewer studies on neutral lipids, phospholipids, and fatty acids as surfactants. Glycolipid biosurfactants, containing one or several sugar units and one to two alkyl or alkenyl chains, are primarily produced by *Candida bombicola*, *Pseudomonas aeruginosa*, and *Bacillus subtilis*, among others. Glycolipids and lipopeptide biosurfactants are the most promising for research, including rhamnolipids (Figure 1a), mannosylerythritol lipids (Figure 1b), sophorolipids (Figure 1c), succinyltrehalose lipids (Figure 1d), and surfactin (Figure 1e). Biosurfactants are increasingly used in various fields due to their sustainability and uniqueness. Understanding their genetic makeup not only helps to reveal their biological synthesis at a molecular level, but also offers potential improvements to the production process. Specifically, overexpression of key genes that encode surfactants could significantly enhance the efficiency of microbial surfactant production [8]. Table 2 summarizes the coding gene of common biosurfactants.

Rhamnolipids, mainly synthesized by *P. aeruginosa*, result from the workings of *RhlA*, *RhlB*, and *RhlC* genes [59]. The *RhlA* gene separates β-hydroxydecanoic acid from the FASII cycle, which forms hydroxyacid esters by joining with another hydroxy fatty acid. *RhlB* then adds a DTDP-rhamnose to create a mono-rhamnolipid, and the *RhlC* gene forms a di-rhamnolipid from the mono-rhamnolipid. *P. aeruginosa*, an opportunistic pathogen, is often reported as a main producer of rhamnolipids. The pathogenic effects of this bacteria can be controlled through genetic engineering in different hosts. For example, the restructured *Escherichia coli (E. coil) pPM RhlAB* and *E. coli pPM RhlABC* can produce single and double rhamnolipids. Using this method, a maximum production of 318.42 mg/L of rhamnolipids has been achieved in an environment with 20% (*v*/*v*) palm manufacturing waste water [60]. By integrating the rhamnolipid synthesis encoding gene *RhlAB* into *Pseudomonas putida* KT2440 and removing the genes of the competitive pathway, research achieved a rhamnolipid production of 19.77 g/L using glucose/glycerol as a mixed carbon source. This result demonstrates the promising potential of KT2440 as a potent microbial cell factory for industrial rhamnolipid production [61].

Sophorolipids, mainly synthesized by oil-soluble yeasts like *Starmerella bombicola*, consist of two sophorose and one fatty acid molecules, showcasing potent surfactant and emulsifying qualities. The *cyp52m1* gene helps in converting unsaturated fatty acids into acyl glycerol, while *ugtA1* and *ugB1* genes are responsible for adding glucose units, potentially modified by the *at* gene. The SL transporter exports sophorolipids, and the *SBLE* gene plays an essential role in sophorolipid maturation. Optimized strains can cut down costs or boost yield [62]. Li et al. recombinant the cellulase gene into ***S. bombicola*** strains, enabling them to degrade lignocellulosic cellulose for sophorolipid production. The concentration of sophorolipids can reach 1.879 g/L when the medium contains 1% regenerated amorphous cellulose [63]. The three transcription factor genes *ztf1*, *leu3*, and *gcl* in *S. bombicola* CGMCC 1576 were knocked out. This action was found to affect the expression of the sophorolipid synthesis genes *sble*, *UGPase*, *ugta1*, and *ugtb1*, resulting in a gradual increase in the yield of SLs. The yield of sophorolipids in the knocked out strain reached 97.44 g/L; compared to the strain without gene knockout, it increased by 50.51% [64].

Mannosylerythritol lipids are synthesized primarily by yeast strains such as *Candida antarctica*, *Candida apicola*, *Pseudozyma hubeiensis*, and *Yukuza tsukubaensis*, which is a sequential process, initiated by the *emt1* gene. This gene encodes a glycosyltransferase which binds mannitol and lysine, and this is the first step in MEL production. Then, *mac1* and *mac2* gene-encoded enzymes connect the fatty acid chain to the glycosyl backbone, and it is a crucial element of MEL production. Next, the *mat1* gene-encoded enzyme may alter or stabilize the MEL molecule, something requiring further research [65]. Lastly, the *mmf1* gene-encoded transposase may transfer the final product outside the cell in the final stages of MEL production. In *Chlamydomonas reinhardtii*, heterologous expression of *mmf1* nearly doubled lipid content [66]. In sum, the joint action of these genes leads to MEL biosynthesis.

Lipopeptide biosurfactants, predominantly produced by organisms such as *B. subtilis*, *Bacillus licheniformis*, and *Micrococcus luteus*, comprise a sequence of amino acids and one to two fatty acids. Surfactin is the most extensively employed among lipopeptides, encoded by the *sfp* genes and the *srfA-A*, *srfA-B*, *srfA-C*, *srfA-C-TE*, and *srfA-TE* genes [67,68]. The *sfp* and *srfA* genes are commonly used. The *sfp* gene helps activate nonribosomal peptide synthetases, and the *srfA* genes code for a complex aiding. Fang Zhang et al. successfully modified the surfactin biosynthetic gene cluster, affecting the transcription level ratio of the *srfA* operon, and increased the production of surfactants. The surfactin amount produced by the modified strain GR167IDS was 311.35 mg/L, which was 10.4 times that of the original strain GR167 [69]. The productivity of the surface protein was successfully enhanced to 2203 mg/L by genetically modifying *B. subtilis* 168. This was accomplished by overexpressing the *sfp* gene and partially eliminating the encoding provided by the *fadE* gene, which subsequently increased the supply of the precursor, acyl-coenzyme A [70]. Additionally, *B. subtilis* 168 was also genetically engineered. The branched fatty acid biosynthesis was enhanced to increase the supply of the precursor acyl-coenzyme A. The transcription of *srfA* was strengthened to shift acyl-coenzyme A from cellular growth to surfactant biosynthesis. The titer of the surfactant was ultimately increased to 12.8 g/L [71].

**Table 2:** Classification of biosurfactants based on chemical properties, bacterial strains, and responsible genes.

Classification	Examples	Bacterial Strains	Responsible Genes
Glycolipids	Rhamnolipids	*P. aeruginosa*	*RhlA*, *RhlB*, *RhlC* [59]
	Sophorolipids	*Torulopsis bombicola*, *S. bombicola*	*cyp52m1*, *ugtA1*, *at*, *SL transporter*, *ugB1*, *SBLE* [62]
	Mannosylerythritol lipids	*C. bombicola*, *Schizonella melanogramma*, *Geotrichum candidum*	*emt1*, *mac1*, *mac2*, *mat1*, *mmf1* [72]
	Trehalose lipids	*Rhodococcus erythropolis*, *Mycobacterium* sp., *Gordonia* sp.	*otsA*, *otsB*, *treY*, *treZ* [73]
	Exopolysaccharide	*Lactobacillus* spp.	*epsABCDE*, *gt*, *Wzx*, *Wzy* [74]
Lipopeptides	Surfactin	*B. subtilis*	*sfp*, *srfA*-A, *srfA*-B, *srfA-*C, *srfA-C-TE*, *srfA-TE* [67,68]
	Lichenysin	*B. licheniformis*	*licAA*, *licAB*, *licAC*, *licAD* [75]
	Iturin	*B. subtilis*	*ItuD*, *ItuA*, *ItuB*, *ItuC* [76]
	Arthrofactin	*Pseudomonas* sp. MIS38	*ArfA*, *ArfB*, *ArfC* [77]
	Polymixins	*Bacillus polymyxia*	*pmxA*, *pmxB*, *pmxC*, *pmxD*, *pmxE* [78]
	Nisin	*Lactococcus lactis* spp.	*nisZBTCIPRKFEG* [79]
Neutral lipids, Phospholipids and Fatty acids	Neutral lipids	*Rhodotorula*, *Rhodosporidium*, *Lipomyces, Trichosporon*, *Candida genera of yeasts*	*ACCase*, *ACL*, *DAG*, *PDAT*, *GPAT*, *LPAAT*, *DGAT* [80,81]
	Phospholipids	*Saccharomyces cerevisiae*	*INO1*, *INO2*, *INO4*, *CHO1*, *CHO2*, *OPI1*, *OPI3*, *PIS1*, *SIN3*, *UME6*, *PSD1/2*, *INM1* [82,83]
	Fatty acids	*P. aeruginosa*, *Corynebacterium lepus*, *B. subtilis*	*accABCD*, *fabD*, *fabH*, *fabB*, *fabF*, *fabG*, *fabA*, *fabI*, *TE* [84]

## 3. Application of Biosurfactants in Medical Sciences

### 3.1. Drug Carrier Components

**Figure 2:**
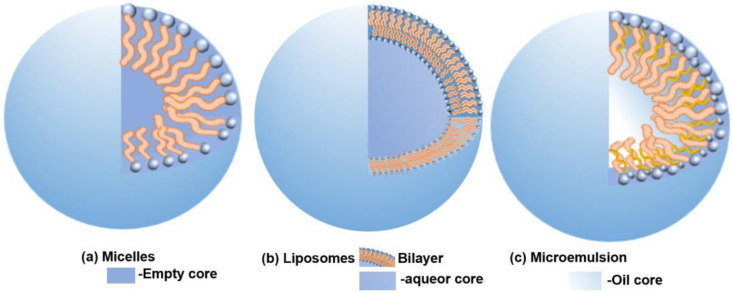
Three forms formed by self-assembly of biosurfactants: (**a**) micelles, (**b**) liposomes, (**c**) microemulsion.

Nanocarriers, distinguished by their progressive drug delivery capabilities, can notably enhance drug bioavailability. Biosurfactants, due to their unique properties, are often employed as vital constituents in drug delivery nanocarriers. In the treatment of multidrug-resistant diseases and cancer, researchers are attempting to incorporate biosurfactants into drug carriers to improve drug delivery efficiency and efficacy, which is currently a popular research topic. At the same time, biosurfactants can also be synergistically administered with other drugs, acting through different mechanisms to improve treatment effectiveness [85]. Their surface activity allows them to interact with cell membranes, which potentially augments the efficacy of drug delivery to targeted cells. Beyond that, the multifunctionality of biosurfactants may act synergistically with drugs to enhance their therapeutic effects. In the process of drug transport, biosurfactants chiefly exist in three forms: micelles, liposomes, and microemulsions [86]. To fully optimize their capabilities as components of nanocarriers (Figure 2), such as micelles, liposomes, and microemulsions, it is imperative to comprehend the interaction mechanisms of biosurfactants and the advantages they offer when incorporated into nanocarriers.

As an example of micelles, surfactin A, a biosurfactant, was utilized as a nanocarrier component for Itraconazole (ITZ), a broad-spectrum antifungal drug. Although ITZ can pose potential side effects due to its pronounced hemolytic activity, the low hemolytic activity of surfactin A could effectively mitigate this risk. When encapsulating ITZ within surfactin A micelles, the direct contact between the drug and red blood cells is restricted, which results in a 53 ± 2.63% reduction in hemolytic activity compared to a similar solution-based concentration and significantly improves the antimicrobial efficacy against *Aspergillus fumigatus*, *Aspergillus niger*, and *Candida albicans* [15]. Therefore, for hydrophobic drugs, using biosurfactants as their carrier components not only improves the drug’s bioavailability and reduces its side effects, but also has a synergistic effect on increasing drug efficacy.

**Figure 3:**
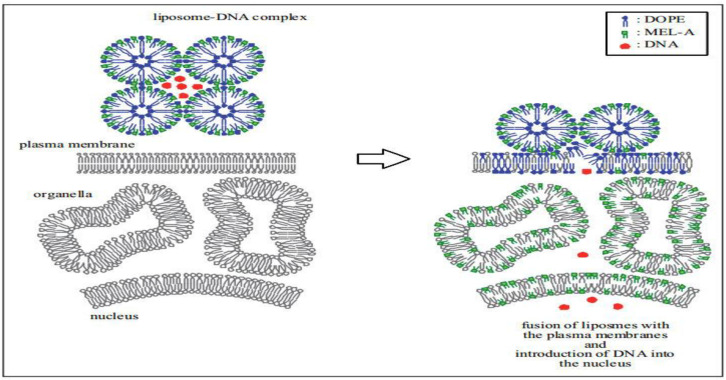
Schematic representation of the first step of the gene delivery pathway mediated by membrane fusion between the nano vectors with MEL-A and plasma membrane of target cells. Figure adapted from [89] with permission of ref. Nano vectors with a biosurfactant for gene transfection and drug delivery, copyright @ Elsevier B.V.

Liposomes are a promising tool for treating hereditary diseases through their ability to deliver foreign genes to target cells. They can transport a wide variety of drugs and are made more stable with the addition of biosurfactants. Together, di-rhamnolipids and dioleoyl phosphatidylethanolamine (DOPE) can create pH-sensitive liposomes that remain stable at a neutral pH but exhibit time-dependent release in a more acidic environment. This allows them to fuse with endosomal membranes and release their contents into the cytoplasm [87]. Niaz and colleagues further illustrated that rhamnolipids enhance liposomes’ functionality, increasing the encapsulation efficiency while boosting their stability and slowing nisin release. This improves antimicrobial activity [88]. Significantly, MEL-A possesses high binding affinity for human immunoglobulin G (HIgG). When incorporated in nanocarriers, it binds lectins on target cells, initiating membrane fusion. This process allows for efficient DNA transportation into target cell nuclei (Figure 3) [89]. The unsaturated fatty acid in MEL-A can affect its interaction with HIgG. This then affects DNA encapsulation, membrane fusion, and DNA release during gene transfer. An unsaturated fatty acid ratio of 21.5% achieves higher gene transfer efficiency compared to ratios of 46.5% and 9.1%. Biosurfactants, which bind to specific target cells, can improve drug therapy accuracy and are important in drug delivery [90,91]. Adding 10% MEL-A into anticancer liposomes containing betulinic acid (BA) increases destruction of mitochondrial membrane potential in HepG2 cells. It also interrupts RNA and protein creation in these cells, enhances the anticancer activity of BA-containing liposomes, prevents cells from entering the S phase, and increases HepG2 cells’ apoptosis rate by 4.44–7.8% [92].

Considering microemulsions, the loading of etoposide in the submicron microemulsion prepared with acidic form sophorolipids as drug carrier components was increased by 112.6% compared with that prepared with Tween. At the same concentration of 40 μg/mL, the acidic sophorolipid–etoposide microemulsion showed an increase in the apoptosis rate of A2780 cells compared to the Tween–etoposide and etoposide injection groups, with improvements of 46.5% and 60.8%, respectively [17]. MEL-A has significant potential to form water/oil microemulsions without any auxiliary surfactants, with a maximum oil phase percentage of 20%. Its encapsulation capability for the oil phase is on par with that of soybean lecithin [93].

In short, biosurfactants are the main parts of drug carriers, improving drug dissolution, boosting target adherence, increasing drug bioavailability, and avoiding multidrug resistance, which are often seen in common treatments. Including biosurfactants opens the way for crafting innovative and better drug delivery strategies, especially when usual methods do not work well.

### 3.2. Inducing Tumor Cell Death and Differentiation

**Figure 4:**
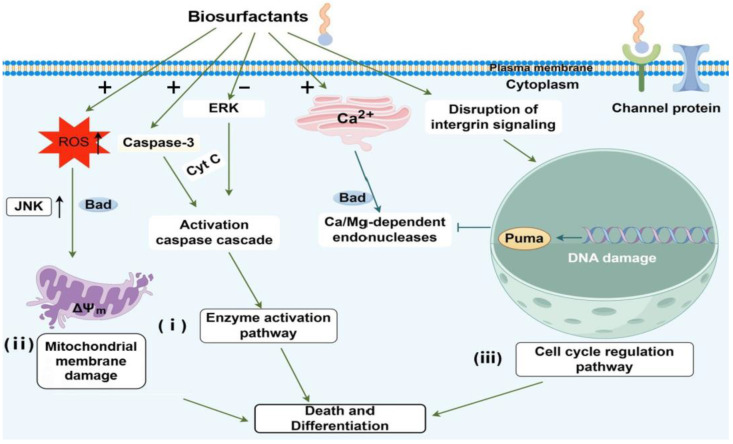
Three pathways of biosurfactant interact with tumor cells to induce differentiation and death. Cyt C, cytochrome; ERK, extracellular signal regulated protein kinase; JNK, c-Jun N-terminal kinase; ROS, reactive oxygen species; Caspase-3, Cysteine-Aspartic Acid Protease 3.

Biosurfactants have diverse biopharmaceutical activities, especially as glycolipid biosurfactants can promote tumor cell death and differentiation to inhibit tumor growth and metastasis. The structure of glycolipids is similar to the membrane components of mammalian cells, i.e., glycosphingolipids and gangliosides [94,95], which are involved in processes such as signaling, oncogenesis, and differentiation. However, there is no clear literature indicating that the ability of biosurfactants to induce tumor cell differentiation is because of a structurally similar reason. Therefore, more research is needed to investigate the mechanism of action of biosurfactants. There are three pathways through which biosurfactants induce differentiation and death in tumor cells (Figure 4).

(I) Enzyme activation pathway: Sophorolipids can induce varying forms of death across different cancer types. In particular, they trigger various forms of death in tumor cell K562, such as swelling, cell content release, cell disintegration, nuclear fragmentation, and nuclear lysis. Lactonic sophorolipids can slow cell growth and potentially trigger programmed cell death (apoptosis) in HepG2 cells via the caspase-3 pathway [18]. Sophorolipid at a concentration of 40 µg/mL can activate caspase-3, increase cytoplasmic Ca^2+^ concentration, activate Ca/Mg-dependent endonuclease pathways, and induce apoptosis in H7402 cells [19].

(II) Mitochondria pathway: Lactonic sophorolipids can generate reactive oxygen species (ROS), modulate mitochondrial membrane potential (Δ*Ψ_m_*), and impact the migration of cancer cells, resulting in the necrosis of lung cancer (A549), breast cancer (MDA-MB231), and mouse skin melanoma cell lines [20]. When B16F10 cells were treated with MEL-B, the increase in ROS levels led to the apoptosis of cancer cells [21]. Nisin is a potent bacteriocin produced by *Lactococcus lactis* subsp. In Hosseini’s study, Nisin (800 µg/mL) significantly reduced cancer cell survival through mitochondrial-dependent apoptosis, shown by increased expression of caspase genes and Bax mRNA levels, as well as elevated caspase-3 and -9 activities, highlighting the apoptosis pathway activation [96].

(III) Cell cycle regulation pathway: Some biosurfactants can inhibit tumor cell proliferation and differentiation, thereby suppressing tumor growth and metastasis. For example, the phosphorylation level induced by MELs is similar to, but not identical to, the effect of the nerve growth factor. MELs trigger the differentiation of rat pheochromocytoma PC12 cells to neuronal cells through an ERK-related signal cascade, leading to the transactivation of the c-jun gene, distinct from the signaling pathway activated by NGF. These findings lay the foundation for utilizing microbial extracellular glycolipids as innovative agents against cancer cells [23]. Four analogs of STL-3 were examined for their ability to inhibit the growth and induce the differentiation of HL-60 human promyelocytic leukemia cells. Results similarly indicate that the ability to inhibit the growth and induce depends on the structure of the hydrophobic moiety of STL-3 [24]. MELs can affect the cell cycle of tumor cells in a concentration-dependent manner. At a MEL concentration of 3–5 mmol/L, only a few cells show signs of apoptosis. In B16 cells exposed to 5 mmol/L MELs for 2 days, the protein kinase PKC was enhanced. Then, B16 cells under 10 mmol/L MELs entered the G1 phase and began apoptosis within 24 h [25].

The actual situation may be more complex. The specific pathways and effects of influence may vary depending on factors such as cell type, type of biosurfactant, and concentration used. Therefore, more in-depth and specific research is needed to target specific biosurfactants and tumor cells. Envisaging their role in the medical field, the potential of biosurfactants is immense. Their ability to selectively perform cell differentiation or cause cell mortality provides a strong foundation for developing more effective and targeted cancer treatments. It could open up new prospects in early detection and intervention of various types of cancer, significantly improving patient outcomes. Thus, future studies should keep exploring the healing potential of biosurfactants. They could be a crucial part of cancer treatments and wider healthcare.

### 3.3. Antibacterial Activity 

**Figure 5:**
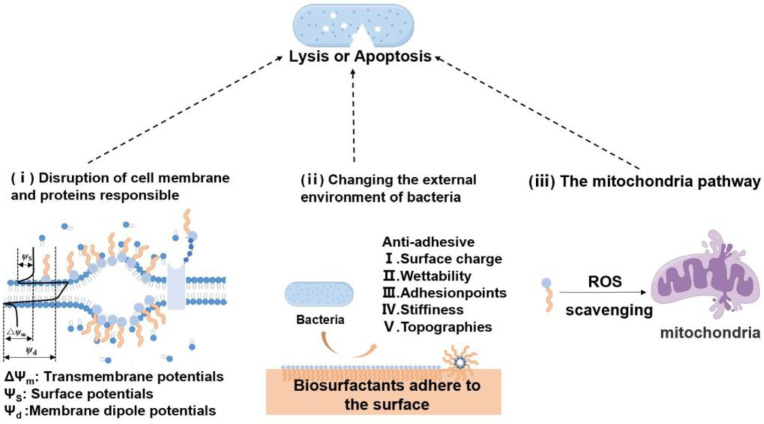
Interactions between biosurfactant and bacterial: (**i**) disruption of cell membrane and proteins responsible for essential function, (**ii**) changing the external environment of bacteria, (**iii**) mitochondria pathway.

Biosurfactants hold significant applications in the field of antibacterial agents, particularly the lipopeptide biosurfactants produced by bacteria, which possess natural antibacterial activity. This innate antibacterial capability lends biosurfactants a wide range of uses within the pharmaceutical field. As shown in Figure 5, these biosurfactants use innovative inhibitory mechanisms, mainly by breaking microbial cell membranes, to achieve antibacterial effects. Increasing biosurfactant concentration may result in the dehydration of the phospholipid bilayer, which may in turn affect membrane functionality and ultimately cause cell death. In addition, certain biosurfactants can affect the adhesion of microorganisms by partitioning the interface of fluid phases with distinct polarities and hydrogen bonding, thereby preventing microbial adhesion to surfaces and tissues and making them more effective at fighting bacteria. While biosurfactants are primarily known for their cleaning properties, they can also interfere with membrane phospholipids or change membrane conductivity [97], making the mitochondria membrane more susceptible to ROS attack and thus more vulnerable, thereby causing damage to microbial cells. Pathogens cannot develop resistance against these multifunction biosurfactants. Therefore, using biosurfactants reduce the risk of pathogen resistance and unwanted side effects. 

(I) Disruption of the cell membrane and proteins responsible for essential function: Surfactin interacts dynamically with lipopolysaccharides (LPS) present in the cell walls of Gram-negative bacteria. At a median effective concentration of 13.75 μmol/L, surfactin significantly diminishes endotoxin levels, tumor necrosis factor-alpha (TNF-α), and nitric oxide in septic shock rats’ plasma. This leads to an increase in survival rates and a decrease in the severity of bacterial infections in septic shock mouse models [27]. Biosurfactants have a concentration-dependent effect on cell membrane damage. For example, lipopeptide biosurfactant surfactants can induce the formation of pores on lipid bilayers at moderate concentrations, while, at high concentrations, detergent-like effects dominate, leading to membrane loss [26].

(II) Modifications in the external environment of bacteria: Biosurfactants diminish interfacial tension, impede the attachment of microorganisms, and act as novel additives for controlling food contamination and plant diseases. Certain biosurfactants, such as MELs, are efficient at reducing the hydrophobicity of solid surfaces, thereby curtailing the attachment of germinating spores and averting the invasive actions of plant pathogenic fungi. Rhamnolipids also exhibit high biofilm inhibitory effects against *Aggregatibacter actinomycetemcomitans* Y4 [28]. The lipopeptide biosurfactants derived from Lactobacillus lactis have demonstrated remarkable results against *Candida*. They possess significant potency—represented by an IC50 value of 5 mg/mL—which indicates their robust impact in inhibiting *Candida* growth. Presenting 50% higher antibacterial activity than the commonly used antifungal drug ketoconazole, these biosurfactants also exhibit superior penetration capabilities, surpassing fluconazole and ketoconazole by 6%. Moreover, they induce cell death in *Candida* by reducing adhesion and interface tension, effectively eliminating pathogens, and permeating the cell wall of *C. albicans* to induce membrane leakage [29].

(III) The mitochondria pathway: Glycolipid and lipopeptide biosurfactants adopt a unique antibacterial approach by increasing the production of ROS. At a concentration of 1/3 MIC (40 mg/mL), rhamnolipid biosurfactants can produce ROS, which helps to kill pathogenic bacteria like *Vibrio cholerae* MTCC 3904 and *Clostridium perfringens* MTCC 450 [30]. Surfactin can increase the production of ROS, which can cause serious damage to the cell wall and the cytoplasm, leading to the death of *ash-branch bacilli* cells [31]. This method not only combats all types of microbes, including antibiotic-resistant bacteria, which common antibiotics struggle to deal with, but also prevents bacteria from developing resistance through damaging their cell wall and cytoplasm.

These biosurfactants curb biofilm formation without hindering pathogenic bacterial growth, and represent a new type of green antibacterial agent against bacteria that has resistance to traditional antibacterial agents. This expands the application range of biosurfactants with antibacterial properties as new functionalized antibacterial agents. Their broad applications include oral infection prevention, plant protection, and food preservation.

### 3.4. Antiviral Activity

Glycolipid and lipopeptide biosurfactants have emerged as promising candidates for antiviral therapeutics, particularly for combating enveloped viruses. The distinct physical and chemical mechanisms of surfactants reduce the likelihood of fostering resistance in drug-resistant pathogens. This stands in contrast to numerous antibiotics, where their high-level toxicity and selectivity can inadvertently enhance the resistance of drug-resistant viral strains. As such, biosurfactants represent a potentially advantageous alternative in the therapeutic landscape.

Biosurfactants demonstrate unique abilities in the modulation of viral behaviors. When surfactants are added to the virus’s outer covering, they trigger a change in how easily substances can pass through it. This is achieved through the creation of channels for ions. This process intensifies at higher surfactant concentrations, leading to disturbances in the membrane system [98]. Under specific conditions, biosurfactants have showcased their potential in inactivating a range of viruses, including herpes viruses, retroviruses, and other enveloped RNA or DNA viruses.

Notably, a cationic lipoprotein derived from the metabolites of the *Haloarchaeon*, *Natrialba* sp. M6, exhibits significant antiviral activity. Its primary mode of action involves the incapacitation of the viral envelope, impeding the virus’s ability to penetrate host cells. Additionally, it inhibits the replication of viruses mediated by RNA and DNA polymerase [32]. This dual-faceted approach underscores the potential therapeutic utility of this biosurfactant.

Sophorolipids, another prominent class of biosurfactants, offer a distinct antiviral mechanism. They possess the ability to dissolve the lipid envelope of SARS-CoV-2, effectively inactivating the virus. Moreover, sophorolipids can work as immunomodulators, attenuating the cytokine storm induced by SARS-CoV-2 infection and abating the detrimental effects of COVID-19 on the human body [33]. Enhanced repurposing of antibacterial peptide biosurfactants has shown promise in treating SARS-CoV-2 infection by reducing viral loads and modulating cellular processes [99].

In summary, biosurfactants present comprehensive multi-tiered strategies against viruses, warranting further exploration of their antiviral capabilities.

### 3.5. Wound Healing and Tissue Repair

Wound healing is an intricate process involving the restoration of damaged tissue and conventionally proceeds through three main phases: inflammation, proliferation, and remodeling. Both attenuation of inflammation and defense against oxidative stress are instrumental throughout the healing process. Inflammation reduction aids in controlling swelling, wound cleansing, and minimizing tissue damage, while antioxidants safeguard cells from damage brought on by oxidative stress, contributing to cellular proliferation and tissue repair. Therapeutically, it is beneficial to utilize substances exhibiting both anti-inflammatory and antioxidant properties.

In the inflammatory phase, biosurfactants, known for their anti-inflammatory and antibacterial properties, can mitigate the inflammatory response by curtailing intracellular inflammatory markers. For instance, the ceramide structure of a biosurfactant variant, MEL-A, facilitates water permeability into intercellular spaces, allowing MEL-A to crystallize within, sustaining intracellular hydration. Intriguingly, MEL-A evidences potential of rectifying cell damage engendered by SDS at a concentration of 1 wt% [100]. Moreover, crude biosurfactants obtained from *Lactobacillus casei* strains have demonstrated antioxidant activity, offering a preventive measure against oxidative stress reactions throughout the wound healing process [34].

In the proliferation stage, biosurfactants further endorse cellular migration and proliferation, synergistically assisting tissue repair. Specifically, an ointment infused with glycolipid biosurfactant derived from *B. licheniformis* SV1 promotes the re-epithelialization and fibroblast cell proliferation at the wound site [35]. Lipopeptide biosurfactants stimulate angiogenesis in Human Umbilical Vein Endothelial Cells. It was established that lipopeptide biosurfactants at a concentration of 300 µg/mL significantly augmented the protein expression of hypoxia-inducible factor-1α (HIF-1α) and vascular endothelial growth factor, accelerating wound healing [11].

In the remodeling phase, biosurfactants aid in the reduction in collagen accumulation at the wound site, thereby fostering tissue remodeling. Rhamnolipids considerably accelerate wound repair and abate collagen accumulation at the injury site [36]. Of significance, di-rhamnolipids and mono-rhamnolipids at non-inhibitory concentrations diminish the expression of IL-8 and CXCL8 in the LPS-stimulated human keratinocyte cell line (HaCaT cells) while enhancing IL-1RA and IL-1RN levels [10]. Thus, di-rhamnolipids and mono-rhamnolipids may eminently wield therapeutic potential to induce anti-inflammatory mediators in diseased skin, modulating the incessant cascade of pro-inflammatory cytokines. Additionally, in later stages, the aforementioned ointment concentrated with glycolipid biosurfactants accelerates collagen deposition at the wound site [35].

Biosurfactants effectively perform therapeutic roles in each distinct phase of wound healing and tissue repair. With anti-inflammatory and antibacterial properties, and abilities to bolster cell growth and attenuate collagen accumulation, they have signaled a new era for wound treatment. While the current research is needed, further investigation is urged to discover additional potential applications. Looking into the future, an exhaustive and in-depth examination of biosurfactants could potentially yield more efficacious and safer therapeutic alternatives for wound management and other medical applications.

### 3.6. Immunomodulatory Effects

**Figure 6:**
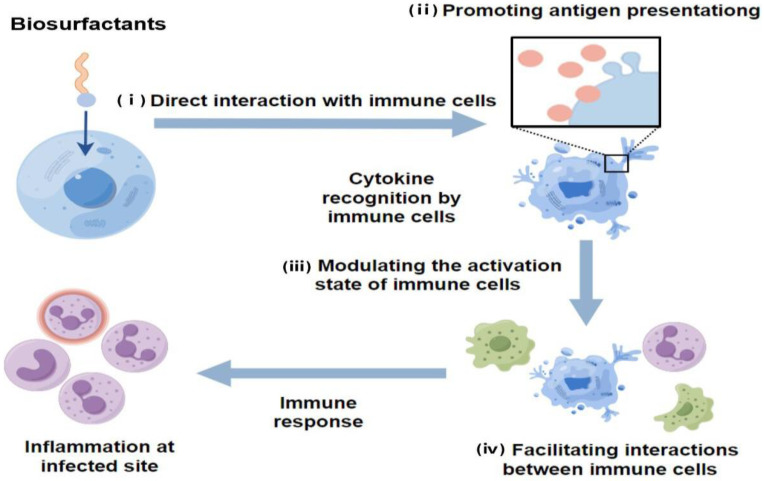
The four pathyways in which biosurfactant affect inflammatory responses and immune regulatory networks (**i**) direct interaction with immune cells. (**ii**) promoting antigen presentation. (**iii**) modulating the activation state of immune cells. (**iv**) facilitating interactions between immune cells.

Biosurfactants interact with receptors on immune cells, affecting how cells communicate internally and regulating their activation, inflammatory responses, and immune regulatory networks. As shown in Figure 6, the pathways through which biosurfactants participate in immune responses include the following: (i) Direct interaction with immune cells: Biosurfactants can directly interact with immune cells, triggering signal transduction pathways and influencing cell activation, differentiation, and migration. (ii) Promoting antigen presentation: Biosurfactants can enhance the solubility and stability of antigens, facilitating their interaction with immune cells. (iii) Modulating the activation state of immune cells: Biosurfactants can regulate the activation state of immune cells, promoting their activation and secretion of immune regulatory molecules. (iv) Facilitating interactions between immune cells: Biosurfactants enhance cell adhesion and aggregation, promoting the formation of immune cell clusters and enhancing the synergistic effect of immune response. Biosurfactants boost the activity of immune cells, especially macrophages, by activating receptors on the cell surface, controlling internal communication pathways, and promoting the production of cytokines. Mycobacterial glycolipid trehalose 6,6′-dimycolate and some glycolipid biosurfactants exhibit intense immune regulatory activity [101]. They can engage with receptors on macrophages, prompting the release of inflammatory cytokines that counteract infections. Moreover, biosurfactants can induce various immune responses, including innate immunity, early adaptive immunity, and both humoral and cellular adaptive immunity, by triggering the production of multiple chemokines and cytokines.

In regulating inflammation, biosurfactants influence the production of inflammatory cytokines by immune cells, including TNF-α, interleukin-1 (IL-1) and interleukin-6 (IL-6). Biosurfactants can suppress excessive inflammatory reactions or promote controlled inflammatory responses, maintaining immune system balance. For example, at non-inhibitory concentrations, di-RL significantly attenuated IL-8 production and CXCL8 expression while increasing IL-1RA production and IL-1RN expression in lipopolysaccharide-stimulated HaCaT cells. Di-rhamnolipids can attenuate the inflammatory response to lipopolysaccharide (LPS) by regulating chemokines and cytokines [10]. Furthermore, glycolipid biosurfactants can stimulate the production of TNF-α, interleukin-1β (IL-1β), and IL-6, thereby enhancing inflammation and targeting intracellular pathogens. On the other hand, surfactin inhibits the expression of MHC-II and co-stimulatory molecules, impairing the antigen-presenting capability of macrophages [37]. A glycolipid biosurfactant (GLB) from *Rhodococcus ruber* IEGM 231 was found to stimulate TNF-α, IL-1β, and IL-6 production, while showing an inhibitory effect on monocyte adhesion. GLBs at concentrations of 10^−2^ to 10^4^ µg/mL had no inhibitory effect on 3H-thymidine in cell cultures. Furthermore, at the maximum GLB concentration of 10 mg/mL, there was hardly any stimulation observed in human lymphocytes. GLBs displayed no cytotoxicity against human lymphocytes, and therefore could be proposed as potential immunomodulating and antitumor agents [38]. These recent findings provide insights into the immunopharmacological potential of biosurfactants in various contexts, including autoimmune diseases and transplantation.

Biosurfactants influence the immune system by affecting immune cell interactions, balancing cytokines and supervising overall immune responses. For instance, MEL-A liposomes have specific interactions with macrophage receptors or a strong attraction to immunoglobulins, making it easier to deliver immune modulators to the suitable immune cells [102]. Moreover, sophorolipids have shown the ability to modulate the immune response by reducing Ig E production in B lymphocyte cell line U266 cells through the downregulation of essential genes involved in Ig E pathobiology [103]. MELs demonstrate anti-inflammatory effects by influencing two main intracellular signaling pathways, which include inhibiting the increase in Ca^2+^ and the phosphorylation of MAP kinase, as well as suppressing the secretion of mast cell inflammatory mediators by inhibiting the molecular mechanism of extracellular membrane fusion soluble N-ethylmaleimide-sensitive factor attachment protein receptor [104].

In summary, biosurfactants significantly influence immune cell activation, inflammatory control, and immune network regulation. By stimulating immune cells, modulating signaling pathways, and regulating cytokine production, the immune function is enhanced or attenuated to maintain immune system balance.

### 3.7. Modifiers and Functional Active Components

Biosurfactants act as modifiers and functional active components due to their unique properties, which is vital role for applications in biomedicine. For example, pulsed UV laser irradiation instigated transformations within sophorolipids, specifically targeting the oleic acid-type molecular component of the sophorolipids. This process gave rise to additional double bonds within the molecule, subsequently leading to the evolution of intense green fluorescence. This discovery broadens the prospective utility of glycolipid biosurfactants within the sphere of drug diagnosis [105]. The use of glycolipid biosurfactants, combined with magnetic nanoparticles, has attracted significant research interest. Acidic sophorolipids have been successfully utilized as surface-stabilizing agents for metal and metal oxide nanoparticles. These acidic sophorolipids interact with the iron oxide surface through their carboxylic groups, generating stable nanoparticle aggregates typically below 100 nm. The resultant nanoparticle aggregates amplify the water solubility and biocompatibility of iron oxide, thus positioning sophorolipids as prospective candidates for in-depth investigation, most importantly as surface capping agents of nanoparticles in biomedical applications [39]. The carboxyl functionality of sophorolipids was successfully modified with nitrodopamine (NDA). The derived SL-NDA exhibited qualities of high stability, non-cytotoxicity, and monodispersity, and was subsequently applied as a surface ligand for iron oxide nanoparticles. This underscores the potential of sophorolipids as robust and innocuous surface coatings, elucidating their potential within biomedical and biotechnological applications [12]. Another noteworthy facet of sophorolipids was highlighted when sophorolipid-capped CdTe Quantum Dot (QD)-treated cells exhibited nearly 100% viability in NIH3T3 cell lines but only 65% viability in MCF-7 cancer cell lines. This differential response underscores the diagnostic as well as therapeutic efficacy of these composites, specifically their ability to selectively kill cancer cells while preserving non-cancerous cells [40]. Lastly, owing to their impressive antibacterial activity, biosurfactants, upon covalent bonding with medical-grade polydimethylsiloxane (PDMS-RLs), retain their inherent properties. This retention extends to reducing biofilm formation and significantly thwarting biofilm development, demonstrating over 2.3 units of reduction against methicillin-sensitive *Staphylococcus aureus*, while maintaining stable biocompatibility. These findings substantiate their remarkable potential as antibacterial agents in medical devices [106].

Biosurfactants, with their unique properties and active ingredients, have shown amazing potential as modifiers of functional ingredients. They enhance the intensity of fluorescence for disease diagnosis through modification, and serve as active modifiers for stabilizing magnetic nanoparticles in magnetic resonance imaging and magnetic hyperthermia research. Therefore, more in-depth research should be conducted on the application of biosurfactants in biomedicine and biotechnology, especially in the exploration of new areas such as acting as modifiers agents in medical devices.

## 4. Challenges and Prospects

Using biosurfactants in medicine has a large amount of promise, but there are also big challenges to overcome. Stability is a significant issue, as biosurfactants are subjected to the impacts of environmental factors such as pH, temperature, and ion intensity. Additionally, the presence and yield of biosurfactants, influenced by microbial growth conditions, could potentially affect their consistency and feasibility in large-scale medical use. As metabolites of microorganisms, the safety of biosurfactants for humans, especially for those with immune deficiencies, requires further research and confirmation.

However, the future projections for biosurfactants in medical applications are extensive and promising. Their advantages as drug carriers, such as increasing the solubility, absorption, and bioavailability of drugs, can assist in optimizing and enhancing clinical treatments. Additionally, the capacity of biosurfactants to induce tumor cell death and differentiation may offer a path for the development of novel anticancer treatments. Antimicrobial and antiviral activities may also provide opportunities for creating new anti-infectious strategies. In regenerative medicine, the efficacy of biosurfactants in promoting wound healing and tissue repair provides new routes for research and application. Additionally, their immunomodulatory function can be potentially used in the treatment of immune-related diseases and in the development of vaccines, further broadening their application in the medical field.

In conclusion, despite the potential limitations and challenges that may be encountered in the application of biosurfactants, the manifold possible modes of their employment in the medical field still suggest substantial future prospects. With in-depth research and gradual technical advances, it is anticipated that biosurfactants will play an increasingly important role in the medical field.

## Figures and Tables

**Table 1:** The application and function of biosurfactants in various fields of medicine.

Application	Function	Reference
Drug Carrier Components	Enhance drug delivery capabilities and bioavailability	[15,16,17]
Inducing Tumor Cell Death and Differentiation	Activate enzyme pathway	[18,19]
	Effect mitochondria pathway	[20,21,22]
	Regulation cell cycle	[23,24,25]
Antibacterial Activity	Disruption of cell membrane and proteins responsible for essential function	[26,27]
	Changing the external environment of bacteria	[28,29]
	Effect the mitochondria pathway	[30,31]
Antiviral Activity	Damage the viral envelope and hinder the virus’s ability to penetrate host cells	[32]
	Dissolve the lipid envelope	[33]
Wound Healing and Tissue Repair	Effect the inflammatory phase	[34]
	Endorse cellular migration and proliferation	[11,35]
	Fostering tissue remodeling	[10,36]
Immunomodulatory Effects	Direct interaction with immune cells Promoting antigen presentation Modulating the activation state of immune cells Facilitating interactions between immune cells	[10,37,38]
Modifiers and Functional Active Components	Modifiers of functional ingredients Broadens the prospective utility of glycolipid biosurfactants within the sphere of drug diagnosis	[12,39,40]

## Data Availability

Not applicable.
